# 

**DOI:** 10.1192/bjb.2023.8

**Published:** 2024-04

**Authors:** Kathryn Weston

**Affiliations:** is founder and Head of Research at Tooled Up Education, Dunstable, UK. Email: kathy@tooledupeducation.com

**Figure:**
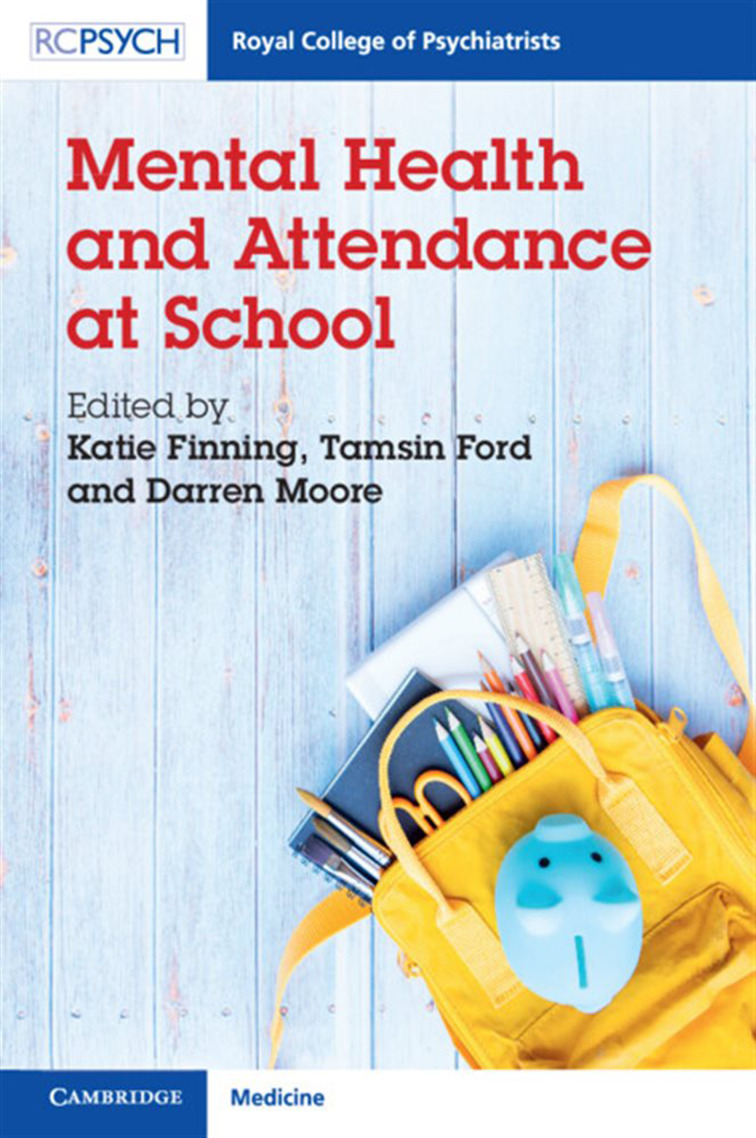

This is arguably the most comprehensive book on school avoidance available. Editors Professor Tamsin Ford, Dr Darren Moore and Dr Katie Finning have ensured that the contributions by the 18 authors reflect a range of perspectives: essential if we are to acknowledge the complex nature of emotion-based school avoidance and develop evidence-based strategies for reducing it.

The book is divided into ten chapters. Initial chapters underline how critical addressing school absenteeism is, given its deleterious impact on a wide range of outcomes for children. It explores associations between absenteeism and mental health problems and addresses when absenteeism becomes problematic. The book's tone is set early on, advocating for compassion when supporting pupils as opposed to punitive approaches.

Dr Moore's chapter on ‘terminology’ reminds readers how important it is to talk about school absence in ways that reflect causal effects and social circumstances. Arguing that a lack of consensus over terminology has been an obstacle, he discusses the debate over terms such as ‘truancy’, ‘school refusal’, ‘school phobia’ and ‘school withdrawal’. He invites us to consider pupils’ motivation for staying away from school as well the range of individual differences contained within existing data. His recommendation that schools strive to achieve consistency by using reliable school attendance measures (which he signposts to) is a good actionable tip.

This book could be described as ‘academic’, but chapters end with summary tips and recommended websites. Those supporting pupils are often not trained in the range of conditions that can contribute to school avoidance, so several chapters describe clinical characteristics of emotional and behavioural disorders and how to support children who exhibit these. Dr Abby Russell's chapter on neurodevelopmental disorders describes common features of a range of disorders and comorbidities, and how school culture can play a role in easing challenges experienced by pupils. A chapter on acquired brain injury (ABI) and school attendance is a welcome and innovative contribution. Recent estimates suggest one child in every classroom will have experienced some kind of brain injury and this can inhibit attendance.

Professor Fazel and Helen Griffith's chapter focuses on finding ways to meet the needs of vulnerable children and describes how multiple adversities can have an impact on attendance and ability to thrive at school. It usefully highlights many of the protective assets associated with children's resilience, including ‘school belonging’.

The book ends with the experiences of a parent whose anxious child did not attend school for a prolonged period. Emma Dalrymple's experience provides insight into the tortuous process that parents can often embark on when seeking help. As Emma says, we are facing ‘a mounting crisis’; this book goes some way to helping us understand the rich variety of factors that contribute to non-attendance, while also highlighting actionable points for schools to implement.

